# Quantitative Angiographic Assessment of Aortic Regurgitation after Transcatheter Aortic Valve Implantation among Three Balloon-Expandable Valves

**DOI:** 10.5334/gh.959

**Published:** 2021-03-19

**Authors:** Hideyuki Kawashima, Rutao Wang, Darren Mylotte, Dariusz Jagielak, Frederico De Marco, Alfonso Ielasi, Yoshinobu Onuma, Peter den Heijer, Christian Juhl Terkelsen, William Wijns, Patrick W. Serruys, Osama Soliman

**Affiliations:** 1 Department of Cardiology, National University of Ireland, Galway (NUIG), Galway, Ireland and CORRIB Corelab and Center for Research and Imaging, IE; 2 Amsterdam UMC, University of Amsterdam, Amsterdam, NL; 3 Department of Cardiology, Radboud University Medical Center, Nijmegen, NL; 4 Department of Cardiac and Vascular Surgery, Medical University of Gdansk, Gdansk, PL; 5 Clinical and Interventional Cardiology Department, IRCCS Policlinico San Donato, San Donato Milanese, IT; 6 Interventional Cardiology Unit, Istituto Clinico Sant’Ambrogio, Milan, IT; 7 Department of Cardiology, Amphia Ziekenhuis, Breda, NL; 8 Department of Cardiology, Aarhus University Hospital, Aarhus, DK; 9 NHLI, Imperial College London, London, GB

**Keywords:** aortic stenosis, balloon-expandable valve, paravalvular regurgitation, transcatheter aortic valve implantation, transcatheter heart valve

## Abstract

**Objectives:**

The aim of the present analysis is to compare the quantitative angiographic aortic regurgitation (AR) after transcatheter aortic valve implantation (TAVI) among three balloon-expandable valves.

**Background:**

Quantitative videodensitometric aortography is an objective, accurate, and reproducible tool for adjudication of AR following TAVI.

**Methods:**

This is a retrospective corelab analysis, independent from industry, of aortograms from patients treated with TAVI using the balloon-expandable Myval transcatheter heart valve (THV) (Meril Life Sciences Pvt. Ltd., India), Sapien 3, and XT THVs (Edwards Lifesciences, Irvine, CA, USA). The study comprised of 108 analyzable aortograms from consecutive patients in a multicenter European registry who underwent Myval THV implantation. The results of quantitative assessment of AR in the Sapien 3 THV (n = 397) and Sapien XT THV (n = 239) were retrieved from a published pooled database.

**Results:**

The Myval THV had the lowest proportion of patients with moderate or severe angiographic quantitative AR (2.8%) compared to the Sapien 3 THV (8.3%; p = 0.049) and Sapien XT THV (10.9%; p = 0.012). Furthermore, the Myval THV had the lowest mean angiographic quantitative AR (6.3 ± 6.3%), followed by Sapien 3 THV (7.6 ± 7.1%) and Sapien XT THV (8.8 ± 7.5%), and it was significantly lower than that of the Sapien XT THV (p = 0.006), but not significantly different from Sapien 3 THV (p = 0.246).

**Conclusion:**

The Myval THV, in comparison with other BEV’s analyzed in our database, showed a lower occurrence of moderate or severe AR after TAVI. These results should be confirmed in prospective cohorts of randomized patients with head-to-head THV comparisons.

Moderate or severe aortic regurgitation (AR) following transcatheter aortic valve implantation (TAVI) has been associated with increased short- and long-term mortality [1]. In the PARTNER 3 trial, among low surgical-risk patients, the rates of moderate or severe AR after TAVI with the balloon-expandable Sapien 3 transcatheter heart valve (THV)(Edwards Lifesciences, Irvine, CA, USA) at 30 days and at one year were similar to those after surgical aortic valve replacement [2]. In the SCOPE 2 trial [3], cardiac death at 30 days (2.8% vs. 0.8%; p = 0.03 for superiority) and 1 year (8.4% vs. 3.9%; p = 0.01 for superiority), and moderate or severe AR at 30 days (10% vs. 3%; p = 0.002 for superiority) were significantly increased in the ACURATE neo THV group compared to the Evolut THV series group. New generation THVs feature sealing skirts or incorporate outer pericardial wrap to minimize AR. The balloon-expandable Myval THV(Meril Life Sciences Pvt. Ltd., India) was granted the CE mark in April 2019. The safety and efficacy of the Myval THV was shown in the MyVal-1 first-in-human trial, with particularly low rates of AR [4].

Quantitative videodensitometric aortography is an objective, accurate, and reproducible tool for adjudication of AR following TAVI [5,6,7,8]. The aim of the present analysis is to compare the quantitative angiographic AR after TAVI among the three BEVs (i.e., Myval THV, Sapien 3 THV, and Sapien XT THV).

This is a retrospective corelab analysis, independent from industry, of aortography from patients treated with TAVI using the Myval THV, Sapien 3 THV, and Sapien XT THV. The CAAS A-valve 2.0.2 (Pie Medical Imaging BV, Maastricht, the Netherlands) was used for assessment of quantitative angiographic AR. This method relies on the angiographic radiopaque density changes after contrast injection in the ascending aorta and its regurgitation (and subsequently density increase) in the left ventricular outflow tract (LVOT) [5,6,7,8]. The ratio between the areas under the two-time density curves of these regions is the AR in percentage. The study included consecutive patients from a multicenter European registry of the Myval THV. The final study cohort comprised of 108 analyzable aortograms from patients who underwent Myval THV implantation according to each participating institution’s heart team recommendation. The results of quantitative assessment of AR in the Sapien 3 THV (n = 397) and Sapien XT THV (n = 239) were retrieved from a published pooled database [8]. The aim of the present analysis is to compare the quantitative angiographic AR following TAVI among the three BEVs.

Continuous variables were reported as mean ± standard deviations. Comparison of LVOT-AR was performed using one-way analysis of variance and 2-by-2 comparisons using the post-hoc Bonferroni test. Stratification of continuous variable regurgitation into categorical variables was performed according to the following pre-determined threshold criteria: 1) none or trace regurgitation (LVOT-AR <6%); 2) mild (6% to ≤17%); and 3) moderate or severe(>17%) [7,8]. The proportion of patients with moderate or severe AR (LVOT-AR >17%) was compared using a chi-square test. A two-sided p value of 0.05 was considered indicative of statistical significance. Statistical analyses were performed with SPSS version 26.0 (IBM, Armonk, New York).

The cumulative frequency curves of LVOT-AR after TAVI for the three BEVs are shown in Panel A. The Myval THV had the lowest proportion of patients with moderate or severe angiographic quantitative AR (2.8%) compared to the Sapien 3 THV (8.3%; p = 0.049) and Sapien XT THV (10.9%; p = 0.012) (Panel B). Furthermore, the Myval THV had the lowest mean angiographic quantitative AR (6.3 ± 6.3%), followed by Sapien 3 THV (7.6 ± 7.1%) and Sapien XT THV (8.8 ± 7.5%), and it was significantly lower than that of the Sapien XT THV (p = 0.006), but not significantly different from Sapien 3 THV (p = 0.246) (Panel C).

**Figure:**
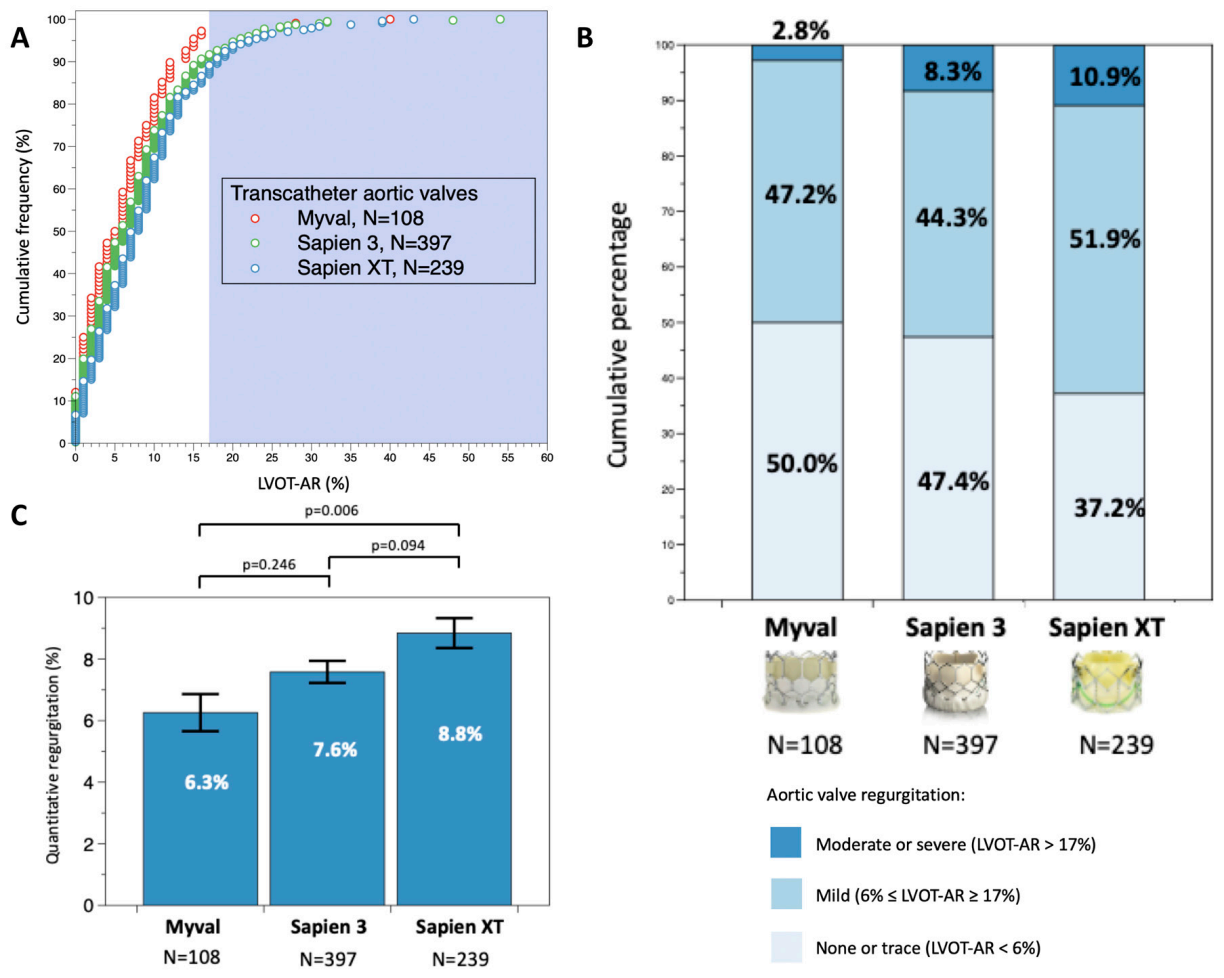
**Panel A:** Cumulative frequency curves of LVOT-AR after TAVI for the three BEVs. **Panel B:** Cumulative percentage of the different degrees of post-TAVI aortic regurgitation among the three BEVs by quantitative angiographic assessment. The shaded background shows the area above 17% of aortic regurgitation, indicating moderate or severe regurgitation. **Panel C:** Comparison of the mean LVOT-AR after TAVI among the three BEVs. Bars denote the mean regurgitation values, and error bars denote standard errors of the mean. LVOT-AR: quantitative aortic regurgitation in the left ventricular outflow tract; TAVI: transcatheter aortic valve implantation; BEV: balloon expandable valve.

The Myval THV, in comparison with other BEV’s analyzed in our database, showed a lower occurrence of moderate or severe AR after TAVI. This could be explained as follow: firstly, the internal skirt of the Myval THV on the valve frame prevents the bioprosthetic valve from inadvertent damage caused by native calcium spicules and also minimizes propensity for AR. Additionally, the external skirt further contributes to minimizing AR by facilitating the plugging of micro-channels at the THV anchor site. Finally, the Myval THV has additional intermediate and extra-large sizes to traditional sizes (20mm, 21.5mm, 23mm, 24.5mm, 26mm, 27.5mm, 29mm, 30.5mm, and 32mm). The availability of nine THV size matrix might eliminate the need for potentially risky under- or oversizing, hence reducing AR after THV implantation. Our findings should be confirmed in prospective cohorts of randomized patients with head-to-head THV comparisons.

The present study has several limitations. First, aortograms were not repeated in the follow-up, since the purpose of the present study was to investigate the acute performance of AR following TAVI using a guidance solely based on aortography. Second, the gold standard for AR assessment is still echocardiography. However, the quantitative videodensitometric aortographic assessment of AR following TAVI by the CAAS A-Valve software has been extensively vetted and validated *in-vitro, in-vivo*, and in the clinical setting [5,6,7,8] and is progressively adopted in TAVI centers with the advent of an online software [9]. Finally, the durability of the three BEVs was not investigated. However, the durability of the Myval THV, Sapien THV and Evolut THV series up to 10 years will be investigated in the ongoing randomized LANDMARK trial (ClinicalTrials.gov NCT04275726, EudraCT number 2020-000137-40) [10]. This trial will provide useful information on the long-term durability of the devices.

## Author Information

Dr. Mylotte is a consultant for Medtronic, Boston Scientific, and Microport.

Dr. Onuma and Dr. Soliman report institutional research grants related to their work as the chairman of cardiovascular imaging core labs of several clinical trials and registry sponsored by industry, for which they receive no direct compensation.

Dr. Wijns reports grants and personal fees from MicroPort, outside the submitted work, and is co-founder of Argonauts, an innovation facilitator.

Dr. Serruys reports personal fees from Biosensors, Medtronic, Micel Technologies, Sinomedical Sciences Technology, St. Jude Medical, Philips/Volcano, Xeltis, and HeartFlow, outside the submitted work.
